# Gender-Egalitarian Attitudes and Assortative Mating by Age and Education

**DOI:** 10.1007/s10680-022-09607-6

**Published:** 2022-04-04

**Authors:** Alessandra Trimarchi

**Affiliations:** Department of Sociology, University of Vienna, Rooseveltplatz 2, 1090 Vienna, Austria

**Keywords:** Mate-selection, Gender-egalitarian attitudes, Assortative mating, Hypogamy

## Abstract

In the last decades, conventional patterns of assortative mating have been challenged by changes in the gender-gap in education. In many countries, educationally *hypogamous* unions (i.e. the woman is more educated than the man) now outnumber *hypergamous* unions (i.e. the man is more educated than the woman). The extent to which such structural changes have also been accompanied by gender egalitarian attitudes has not yet been investigated. This paper fills the gap by focusing on both age and educational assortative mating, using data from wave 1 and 2 of the Generations and Gender Surveys for 6 European countries. I investigate the role of gender-role attitudes of single men and women, measured in the first wave, on their age and educational assortative mating outcomes observed in the second wave. To this aim, I applied multinomial logistic regressions, and used as reference outcome category *remaining single* in the second wave. Compared to non-egalitarian men, I found that men holding gender-egalitarian views are more likely to form hypogamous unions instead of remaining single, in terms of both age and educational assortative mating. Egalitarian women are more likely than non-egalitarian women to form age-hypogamous unions instead of remaining single, but they are less likely to form educationally hypogamous unions. I discuss the implications of these results in relation to the convergence of mating preferences between men and women.

## Introduction

Patterns of human mating behaviour are characterized by dissimilarity and similarity of socio-demographic features between partners. The most conventional mating pattern with regard to the age difference between partners consists in age hypergamy, i.e. the man is older than the woman. With regard to education, typically, the man is at least as educated as the woman. These prevailing patterns have been often considered the outcome of a strong traditional gender division of labour, the male-breadwinner and female homemaker model (Becker, 1991).

Preferences and beliefs about partners have been shaped by people’s expectations for men and women in society (Goldscheider et al., 2009). Historically, the societal norm that the man should be the partner with more socio-economic resources explains the high incidence of age- and educational-hypergamy (Buss et al., 2001; Esteve et al., 2012; Frye & Urbina, 2019). Yet over time, personal choice has become more important as a determinant of partner selection: unions are increasingly viewed in terms of individual qualities rather than social roles (Goldscheider et al., 2009). In many Western societies, the increasing role of personal choice in partner selection has been accompanied by structural changes. Due to the reversal of the gender-gap in education, the proportion of highly educated women on the mating market now exceeds the proportion of highly educated men (Van Bavel, 2012). As previous studies have shown, while homogamy remains steady, hypergamy has decreased (Grow & Van Bavel, 2015; Esteve et al., 2016; De Hauw et al., 2017). These developments represent a challenge for individuals preferring to be in a traditional union where the man is more educated than the woman.

The fact that women of reproductive ages now acquire higher education than men has also enhanced gender-equality in other aspects, for instance with respect to income potential and labour market participation (Klesment & Van Bavel, 2017). In general, the diffusion of gender-egalitarian attitudes affects the composition of mating markets, both in quantitative and in qualitative terms. Over time, mating markets have increasingly been filled with men and women who hold gender-egalitarian attitudes. These attitudes may be manifested on at least two levels. First, in beliefs that regard the private sphere, particularly concerning the relationship between the man and the woman within the couple (Goldscheider et al., 2015). Second, in beliefs concerning the public sphere, i.e. gender equality at the societal level, which relates to the role of men and women in society, e.g. in the labour market (Goldscheider et al., 2010).

Scholars have been interested in the diffusion of gender-egalitarianism because of the links with family outcomes, such as couples’ stability and realized fertility. Existing studies show that, at the individual level, gender-egalitarian attitudes are negatively associated with dissolution rates and positively associated with fertility (Aassve et al., 2015; Goldscheider et al., 2015; Kaufman, 2000). Another aspect that is often mentioned in relation to couples’ stability and fertility is the level of dissimilarity and similarity of partners’ socio-demographic characteristics (Burgess & Wallin, 1943; Hart, 2019; Van Houdt & Poortman, 2018; Voas, 2003). In the past, educationally hypogamous couples, i.e. unions formed by highly educated women and lower educated men, were considered at higher risk of union dissolution and less fertile relative to the educationally hypergamous couples, i.e. unions where the man is more educated than the woman (Van Bavel et al., 2018). Recent studies show that, in some contexts, hypogamy, relative to hypergamy, is not necessarily associated with a higher dissolution risk (Schwartz & Han, 2014; Theunis et al., 2018) or lower fertility (Nitsche et al., 2018; Trimarchi & Van Bavel, 2020). These findings could be explained by selection processes taking place before hypogamous unions are formed, which have ensuing positive effects on family outcomes. I argue that partners’ gender-egalitarian attitudes may be the missing link between the formation of hypogamous unions and family related outcomes. It is likely that those unions are increasingly formed by partners who hold gender-egalitarian attitudes, which in turn favour couples’ stability and fertility. Despite the relevance of selection processes occurring before entry into unions, there is hardly any research on this topic, and the causal link between gender-egalitarian attitudes and union formation patterns remains under researched.

I aim to fill this gap by focusing on singles individuals and their partnership formation processes, controlling for their gender-egalitarian attitudes. To this end, I use two waves of the Generations and Gender Surveys (GGS) for six European countries. I select men and women who are single in the first wave, and examine how their union formation patterns, observed in the second wave, relate to their gender-egalitarian attitudes, which were measured in the first wave. Results show that men who hold egalitarian attitudes are more likely to form hypogamous unions, i.e. where the man is younger or less educated than the woman, relatively to their non-egalitarian peers. This finding, instead, does not always hold for egalitarian women.

## The Gender Revolution and Partners’ Selection Processes

The Gender Revolution as conceived by Goldscheider et al. (2015) comes in two phases. The first phase consists in an increasing participation of women in the public sphere. In particular, it regards the increasing share of highly educated women and increasing female labour force participation. These processes have been associated with a weakening of family life, i.e. higher divorce rates, diffusion of cohabitation and non-marital childbearing, postponement of motherhood and high levels of childlessness (Van De Kaa, 1987; Esping-Andersen, 2009). A second phase of the Gender Revolution is envisioned in the private spheres of home and family, referring to the larger involvement of men in these domains. The sequence of the two phases of the Gender Revolution implies a new gender-balance within the private and the public sphere towards more equal relationships between men and women (Goldscheider et al., 2015).

Within this framework, individuals’ attitudes and their changes over time play a key role in the societal adaptation process to structural changes, favouring the development of new behaviours (Thornton & Young-DeMarco, 2001; Golscheider et al., 2015; Zentner & Eagl, 2015). Gender-norms refer to views held by individuals regarding the roles men and women should play in society, for instance with respect to the division between paid and unpaid work. The attitudes formed in the older, more gender-unequal regimes may still hold during the Gender Revolution, especially for those strata of the population more reluctant to the new gender structure (Davis & Greenstein, 2009; Goldscheider et al., 2015; Rindfuss et al., 1996). As people adapt to the new regime, also due to the increasing participation of women into higher education and labour force, new patterns emerge that may affect demographic outcomes, namely union formation, dissolution and fertility (Esping-Andersen & Billari, 2015; Goldscheider et al., 2015).

### Mating Markets and Mating Preferences

In theories of mating behaviour, the concept of mating market synthesizes two aspects of the mating process: preferences and constraints (Oppenheimer, 1988; Grow & Van Bavel, 2015; for an overview of the market approach to partner’s selection, see Van Bavel, 2021). Individuals look for mates with specific attributes and preferred qualities, but they also face constraints when choosing from the pool of ideal partners. Given the constraints, men and women prefer mates whose attributes they believe will facilitate their well-being in their lives’ outcomes (Zentner & Eagly, 2015: 329).

Changes in gender norms may affect partners’ search patterns and preferences, especially among individuals who are looking for a long-term companion with whom to have children (Meeussen et al., 2019). To fulfil their own family goals, people may be more inclined to choose a partner who supposedly shares similar family goals (Voas, 2003). Typically, researchers interested in studying this topic, due to lack of information, are not completely aware of the composition of the entire mating markets including all potential mates (Van Bavel, 2021). Thus, to study partner selection processes, assumptions on men and women’s mating preferences are required. Previous research on mating preferences has shown that age and education are among the main attributes valued in potential mates (Buss et al., 1990). The present study also focuses on these two attributes, and examines the union formation outcomes in terms of age and educational assortative mating.

### Mating Preferences and Gender-Roles

According to the socio-cultural framework that addresses men’s and women’s mating preferences, these are shaped by individuals’ gender-egalitarian attitudes and the level of gender equality where individuals socialize (Eagly & Wood, 1999; Zentner & Eagly, 2015). In traditional male-breadwinner societies (where men are mostly working in paid jobs and are mainly responsible for the household’s income, whereas women take care of household work), individuals prioritize attributes that maximize the traditional division of labour between partners (Becker, 1991). When the male-breadwinner model holds, men tend to prefer younger and less educated women, who supposedly are less labour-market oriented, whereas women tend to prefer older men with high level of education and high-income potential (Buss, 2000; Buss et al., 1990).

In parallel with partners’ educational differences, age differences also affect power dynamics between the genders, both within couples and in society. Greater gender equality is expected to be associated with lower age-differences (Bozon, 1991). Age hypergamy (i.e. the man is older than the woman) has been linked to limited opportunities for women, inequality and patriarchy. In general, the younger partner is considered to be weaker within the couple because of fewer socio-economic resources and less life experience (Kolk, 2015).

In more gender-traditional societies, both educational hypogamy (i.e. couples where the woman is more educated than the man) and age hypogamy (i.e. unions where the woman is older than the man) are considered *unconventional* or *non-traditional* pairings. Individuals forming these unions may be socially sanctioned because they violate gender norms (Blossfeld & Timm, 2003), they may be considered a threat among their social network (Brines, 1994; Frye & Urbina, 2019).

The shift towards a more gender-equal society observed in recent decades has had implications in relation to ideal characteristics of partners (Goldscheider et al., 2009). Since the 1990s, men increasingly place more importance on women’s earnings potential. Women, conversely, have started to place less importance on men’s earnings (Buss, 2000; Buss et al., 2001; Meeussen et al., 2019; Press, 2004; Zentner & Eagly, 2015). This process might also have been fuelled by structural changes in the composition of mating markets. Mating markets have been increasingly filled with highly educated women, who also participate in the labour market (De Hauw et al., 2017; Grow & Van Bavel, 2015), which might have had positive effect on the overall diffusion of gender egalitarian attitudes (Esping-Andersen & Billari, 2015; Goldscheider et al., 2015).

Still, the causal direction between people’s gender-role attitudes and partner selection at the individual level has remained unexplored. It is not yet clear whether individuals who are attached to more gendered roles act differently from their gender-egalitarian counterpart at the time of partner selection. To understand these mechanisms, it is important to disentangle the effect of attitudes on behaviours, given that behaviour may also influence attitudes. More egalitarian views of the relationship between partners may derive from previous gender-stereotypical partnerships that were not successful. Alternatively, attitudes become more traditional after an unconventional union type was experienced as a failure.

## Research Hypotheses

In this study, I argue that individuals’ who hold gender-egalitarian attitudes are more likely to enter into hypogamous unions relative to individuals who do not hold gender-egalitarian views. To test this thesis, I focus on single men and women and I look at whether those egalitarians are more inclined to form hypogamous unions, rather than remaining single, relatively to their non-egalitarian counterparts. The theoretical argument is that individuals who hold gender-egalitarian attitudes are less attached to a traditional division of labour between partners. I test this argument separately for men and women, focusing on two of the main attributes that play a role in mate selection, i.e. age and education. With regard to the age pattern, I expect that men and women who hold egalitarian views will be more likely to form unions where the woman is older than the man, relatively to non-egalitarian individuals (*H*1). With regard to educational assortative mating, I expect that lower educated men and higher educated women who hold egalitarian attitudes will be more likely to form unions where the woman is more educated than the man, relatively to their non-egalitarian peers (*H*2).

## Data and Methods

### Generations and Gender Surveys (GGS) and Sample Selection

The GGS are part of a wider program whose aim is to improve the knowledge of macro–micro factors that affect the relationships between generations and genders (http://www.ggp-i.org/). The surveys include individuals between 18 and 79 years old, and deal with different topics, such as: fertility and partnership histories, the transition to adulthood, economic activity, care duties, and attitudes.

The GGS are the most recent available large-scale panel and internationally comparable demographic surveys available to date and they are characterized by an independent sample of men and women who were interviewed separately (Gauthier et al., 2018; Vikat et al., 2007). To determine the association between attitudes and union formation behaviour, longitudinal data are necessary. Among all the GGS, data of Austria, Bulgaria, France, Georgia, Hungary, and Poland, have been chosen for the availability of two survey-waves and the availability of the variables necessary to the aim of this paper. The first wave took place at different times for these countries (2004–2011) and the second wave has been carried out 3 (or 4) years later. Table 1 synthesizes information of the survey for each country considered.

**Table 1:** Details of wave 1 and 2 of the GGS samples considered. *Source*: https://www.ggp-i.org/data/methodology/ (consulted on the 13th December 2019)

	Austria	Bulgaria	France	Georgia	Hungary	Poland
Wave 1 (years of interview)	2008–2009	2004	2005	2006	2004–2005	2010–2011
Wave 2 (years of interview)	2012–2013	2007	2008	2009	2008–2009	2014–2015
Percentage of wave 1 sample interviewed in Wave 2 (panel stability) (%)	78	73	88	83	83	62

To answer the research question of this study, I selected single respondents (18–45 years old) in the first wave. From a starting sample of 7606 respondents, individuals were dropped from the sample because of inconsistencies between the first and second waves in reporting the sex and year of birth (*n* = 279), or because formed same-sex couples (*n* = 13), which are not the focus of this paper. Overall, the sample consists of 3693 men and 3621 women.

### Independent Variables

The main independent variables are measured in the first wave and concern gender-egalitarian attitudes at the couple and societal level, in line with previous studies (Aassve et al., 2014; Davis & Greenstein, 2009). I have considered three statements for which the respondent expressed his or her agreement, answering on a five-point scale ranging from *strongly agree* to *strongly disagree*. The statements are the following: “In a couple it is better for the man to be older than the woman” (1); “If the woman earns more than the partner, it is not good for the relationship” (2); “When jobs are scarce men have more right to the job than women” (3). The first two statements indicate gender-egalitarian attitudes within the couple, whereas the third one is an indicator of expected gender-role within society.

A score of 1–2 has been coded as “Non-egalitarian”, a score of 3 as “Neutral”, and a score of 4–5 “Egalitarian”. Each statement relates to different aspects of being gender-egalitarian and this is the reason to keep them separately. As previous scholars noted, attitudes regarding egalitarianism in the private sphere may lead to different demographic outcomes than attitudes regarding the societal sphere (Goldscheider et al., 2010). Additionally, it is relevant to distinguish between couple-level gender-role attitudes and society-level because of the two stage of the Gender Revolution. Gender-egalitarian attitudes related to women’s role in the public sphere are more diffused than equality in the relationships between men and women in the private sphere (Goldscheider et al., 2015).

Following previous studies (Goldscheider et al., 2009; Kaufman, 2000; Sassler, 2010), in addition to measures about gender-egalitarian attitudes, I included control variables that may affect respondents’ union formation: marital history, number of children, age, and educational attainment. It should be noted that about 20% of individuals in the analytical sample has experienced an educational upgrade between the two waves, excluding these individuals from the analyses does not alter the conclusions reported here. Table 2 shows the independent variables for both male and female samples.

**Table 2:** Sample description: Independent variables measured in wave 1. *Source*: Own calculations on GGS data

	Men	Women
	%	%
*Educational level*^*a*^		
Low	14.11	10.05
Medium	61.22	50.15
High	24.67	39.80
*Union history*		
Never in union	84.46	65.81
First union	12.75	27.87
Higher order union	2.79	6.32
*Gender attitudes: age*		
Non-Egalitarian	40.45	41.26
Neutral	35.58	33.11
Egalitarian	23.26	24.97
Unknown	0.70	0.66
*Gender attitudes: earning*		
Non-Egalitarian	22.83	23.67
Neutral	32.63	28.69
Egalitarian	43.33	46.29
Unknown	1.22	1.35
*Gender attitudes: jobs scarce*		
Non-Egalitarian	38.29	18.12
Neutral	25.05	14.50
Egalitarian	36.01	66.69
Unknown	0.65	0.69
Age (median)	26	28
Number kids (mean)	0.2	0.6
*Country*		
Austria	10.29	11.74
Bulgaria	23.12	19.52
France	9.10	12.62
Georgia	18.82	18.39
Hungary	20.66	18.06
Poland	18.01	19.66
*N* total	3693	3621

^a^The level of education refers to wave 2 because it is when educational mating can be measured

### Dependent Variables

The dependent variable is the respondent’s union outcome observed in the second wave, combining co-residential and non-residential unions. In GGS surveys, co-residential unions are considered as such only if they lasted for at least 3 months (Vikat et al., 2007). Robustness checks have been run considering only co-residential unions as outcome variable, results remain substantially the same.

The unions formed have been classified according to the age and educational level of the respondent’s partner. It is not possible to account for unions that have been formed between waves and that did not survive up to the second wave. For the majority of countries considered, partner’s age and education are available only if, at the time of interview, the respondent declares to have a partner.

With regard to the age difference between partners the categories of the outcome variable are: (1) homogamy (defined as an age difference lower than 2 years); (2) hypergamy (i.e. male older 2 years or more); (3) hypogamy (i.e. female older 2 years or more); (4) not partnered in wave 2 (i.e. remained single). Robustness checks have been carried out by considering as homogamous unions those with an age difference of maximum 1 year or 3 years. In both cases, results remained substantially in line with what is presented here.

The second outcome variable is educational assortative mating. Respondents and their partners are grouped into three levels of education (low, medium, high), collapsing categories from the International Standard Classification of Education (ISCED 1997). The first group includes those who completed primary plus lower secondary school (at least 8 years of schooling, ISCED 0, 1, and 2). The medium category consists of individuals who completed the upper-secondary and a post-secondary level (ISCED 3 and 4). Finally, highly educated individuals got a bachelor/master/PhD degree (ISCED 5 and 6).

The variable of educational assortative mating has four categories: homogamous union, where both partners have the same level of education (1); male hypergamy, where the man is more educated that the woman (2), female hypogamy, where the woman is more educated than the man (3), remaining single (4). To check whether the results are sensitive to the definition of the outcome variable, I have also constructed a dependent variable which indicates the level of education of respondents’ partners in the second wave. Table 3 shows the distribution of the outcome variables.

**Table 3:** Sample description, outcome variables (measured during second wave). *Source*: Own calculations on GGS data

Outcome variables	Men		Women	
	%	*N*	%	*N*
*Age difference*				
Homogamy	7.28	269	7.68	278
Hypergamy: male older	16.08	594	18.75	679
Hypogamy: female older	3.44	127	4.11	149
Not partnered	68.24	2520	66.20	2397
Not available	4.96	183	3.26	118
*Educational assortative mating*				
Homogamy	17.30	639	17.56	636
Hypergamy	7.85	290	4.81	174
Hypogamy	4.71	174	8.70	315
Not partnered	68.24	2520	66.20	2397
Not available	1.90	70	2.73	99
*Partner's education*				
Low	7.28	269	5.36	194
Medium	13.64	504	14.77	535
High	8.94	330	10.94	396
Not partnered	68.24	2520	66.20	2397
Not available	1.90	70	2.73	99

### Analytical Strategy

To estimate the effect of gender-role attitudes measured at time *t*_1_ (wave 1 of GGS) on the likelihood that a single man or woman enters into a specific type of union at time *t*_2_ (wave 2 of GGS), I apply a multinomial logit model (Agresti, 2002). Logit models for nominal response variables pair each type of union with a baseline category, which in this case is being single. Formally, it can be written:

$$\log \frac{{\pi_{j} \left( x \right)}}{{\pi_{{J = {\text{single}}}} \left( x \right)}} = \alpha_{j} + \beta_{j}^{^{\prime}} x$$

The regression coefficients are estimated via the maximum likelihood method and a stepwise modelling procedure. Note that, due to missing values in the dependent variables (see Table 3), in models analysing the age difference between partners I had to drop 301 individuals (183 men, and 118 women). In models analysing educational assortative mating, overall 169 individuals (70 men, and 99 women) were dropped. Detailed results of the full models are reported in the "Appendix".

## Results

In this section I show the results obtained from the multinomial regression models, and I, especially focus on the effects of gender-egalitarian attitudes on the likelihood to form *hypogamous* unions. Overall, I found similar results for men and women with regard to the association between gender-egalitarian attitudes and hypogamous unions in terms of age difference between partners. With regard to the educational pairing outcome, findings tend to differ between men and women.

### Gender Egalitarian Attitudes and Age Difference between Partners

**Fig. 1:**
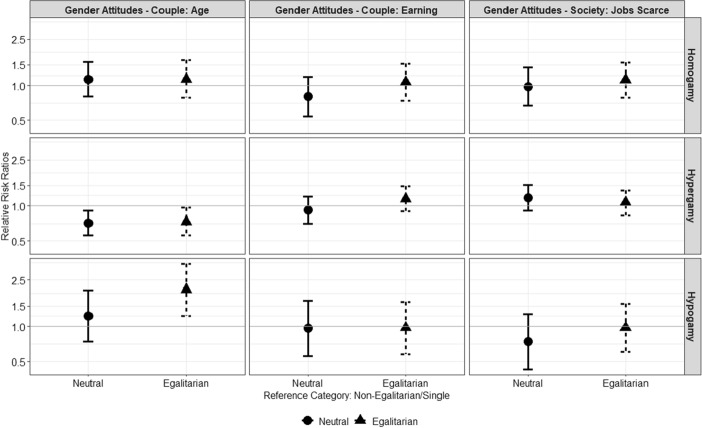
Relative risk ratios and 95% confidence intervals for gender-egalitarian attitudes as predictors of men’s entry into unions by partner’s age (homogamy = partner has similar age, hypergamy = man is older, hypogamy = woman is older)

Figure 1 shows the relative risk ratios for the effect of gender-egalitarian attitudes for the probability to enter a homogamous, hypergamous or hypogamous union, relative to remain single. Full results of the multinomial regression model are shown in Table 4 of the "Appendix".

**Table 4:** Estimated coefficients and 95% confidence intervals for predictors of men’s entry into unions by partner’s age (homogamy = partner has similar age, hypergamy = man is older, hypogamy = woman is older)

Age assortative mating in wave 2 (Ref. single)	Coef.	Std. err.	95% Conf. int	
*Age Homogamy*				
Age	− 0.04	0.09	− 0.22	0.13
Age squared	0.00	0.00	0.00	0.00
Educational attainment (Ref. medium)				
Low	− 0.47	0.25	− 0.95	0.01
High	0.47	0.15	0.17	0.77
Number of children	0.35	0.15	0.05	0.65
Respondent union history (Ref. never in union)				
First union	0.25	0.27	− 0.27	0.77
Higher order union	0.27	0.49	− 0.70	1.24
Gender attitudes couple: age (Ref. non-egalitarian)				
Neutral	0.12	0.17	− 0.21	0.46
Egalitarian	0.13	0.19	− 0.24	0.50
NA	0.31	0.85	− 1.36	1.98
Gender attitudes couple: earnings (Ref. non-egalitarian)				
Neutral	− 0.22	0.20	− 0.61	0.17
Egalitarian	0.07	0.19	− 0.30	0.43
NA	− 0.78	0.81	− 2.37	0.80
Gender attitudes society (Ref. non-egalitarian)				
Neutral	− 0.02	0.19	− 0.40	0.35
Egalitarian	0.11	0.18	− 0.24	0.45
NA	0.38	0.83	− 1.24	2.00
Country (Ref. Bulgaria)				
Georgia	− 0.23	0.23	− 0.68	0.23
France	0.64	0.25	0.15	1.13
Hungary	0.23	0.22	− 0.19	0.65
Austria	0.83	0.23	0.38	1.28
Poland	− 0.54	0.24	− 1.01	− 0.08
Constant	− 0.69	1.23	− 3.09	1.71
*Age Hypergamy*				
Age	0.37	0.06	0.25	0.48
Age Squared	− 0.01	0.00	− 0.01	0.00
Educational attainment (Ref. medium)				
Low	− 0.08	0.14	− 0.36	0.20
High	0.12	0.12	− 0.11	0.34
Number of children	0.21	0.10	0.02	0.40
Respondent union history (Ref. never in union)				
First union	0.60	0.16	0.29	0.92
Higher order union	0.41	0.29	− 0.16	0.98
Gender attitudes couple: age (Ref. non-egalitarian)				
Neutral	− 0.34	0.12	− 0.58	− 0.10
Egalitarian	− 0.31	0.14	− 0.58	− 0.04
NA	0.01	0.61	− 1.18	1.21
Gender attitudes couple: earnings (Ref. non-egalitarian)				
Neutral	− 0.08	0.14	− 0.35	0.19
Egalitarian	0.14	0.13	− 0.11	0.39
NA	− 0.59	0.53	− 1.64	0.45
Gender attitudes society (Ref. non-egalitarian)				
Neutral	0.16	0.13	− 0.10	0.42
Egalitarian	0.06	0.13	− 0.19	0.31
NA	0.86	0.53	− 0.18	1.90
Country (Ref. Bulgaria)				
Georgia	0.12	0.15	− 0.17	0.41
France	0.31	0.19	− 0.06	0.67
Hungary	− 0.16	0.15	− 0.46	0.14
Austria	0.86	0.17	0.53	1.18
Poland	− 0.86	0.18	− 1.22	− 0.50
Constant	− 6.41	0.85	− 8.09	− 4.74
*Age Hypogamy*				
Age	0.08	0.12	− 0.15	0.31
Age Squared	0.00	0.00	− 0.01	0.00
Educational attainment (Ref. medium)				
Low	0.44	0.26	− 0.07	0.96
High	0.55	0.22	0.11	0.98
Number of children	0.25	0.16	− 0.06	0.56
Respondent union history (Ref. never in union)				
First union	0.61	0.32	− 0.01	1.23
Higher order union	1.39	0.43	0.55	2.24
Gender attitudes couple: age (Ref. non-egalitarian)				
Neutral	0.21	0.26	− 0.30	0.72
Egalitarian	0.73	0.26	0.21	1.24
NA	0.57	1.14	− 1.66	2.80
Gender attitudes couple: earnings (Ref. non-egalitarian)				
Neutral	− 0.04	0.28	− 0.59	0.51
Egalitarian	− 0.03	0.27	− 0.56	0.49
NA	− 0.76	1.09	− 2.89	1.36
Gender attitudes society (Ref. non-egalitarian)				
Neutral	− 0.30	0.28	− 0.85	0.24
Egalitarian	− 0.03	0.25	− 0.51	0.45
NA	NA	NA	NA	NA
Country (Ref. Bulgaria)				
Georgia	− 0.21	0.34	− 0.88	0.45
France	0.57	0.35	− 0.12	1.26
Hungary	0.38	0.30	− 0.20	0.96
Austria	0.84	0.33	0.19	1.48
Poland	− 0.95	0.40	− 1.74	− 0.17
Constant	− 3.79	1.67	− 7.07	− 0.52
Log likelihood	− 2801			
*N*	3510			

According to *H*1, single egalitarian men are more likely than single non-egalitarian men to form age-hypogamous unions. In line with this hypothesis, I found that the estimated odds of being in a hypogamous union instead of remaining single are almost two times (exp(0.73)) higher for egalitarian men who disagree with the statement “*If the woman is older than the man is bad for a couple relationship*”, relative to non-egalitarian men, who agreed with that statement. Additionally, egalitarian men who disagreed with that statement are less likely to form age-hypergamous unions instead of remaining single, relative to the non-egalitarian men. There is no evidence of a statistically significant association between the other two statements indicating gender-egalitarian attitudes and the likelihood to form homogamous, hypergamous or hypogamous union relative to remaining single.

**Fig. 2:**
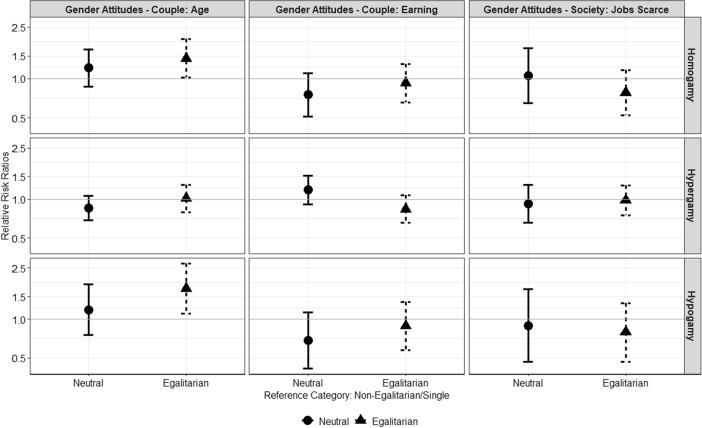
Relative risk ratios and 95% confidence intervals for gender-egalitarian attitudes as predictors of women’s entry into unions by partner’s age (homogamy = partner has similar age, hypergamy = man is older, hypogamy = woman is older)

Figure 2 shows results obtained with the women sample. In line with hypothesis *H*1, women who hold egalitarian attitudes regarding the statement about the age difference between partners have about 1.7 (exp(0.55)) higher estimated odds to form an age-hypogamous union instead of remaining single, relative to non-egalitarian women. Egalitarian women are also more likely to form age-homogamous unions instead of remaining single, relative to the non-egalitarian counterparts. The difference between egalitarian and non-egalitarian women does not emerge considering the other statements, and other unions’ outcomes.

Overall, results show an overlap between attitudes regarding partners’ age differences and the actual type of age-related mating pairing individuals constitute. The findings related to the age-difference between partners support the idea that individuals, both men and women, hold gender attitudes which are consistent with their behaviour.

With regard to the effect of relevant control variables (see Tables 4 and 5 in "Appendix"), I found that both men and women who have experienced already one or multiple unions at the time of the first wave have higher odds to be in a partnership instead of remaining single relative to individuals who have never been partnered. Additionally, for men, having one additional kid increases the odds of partnering with a woman of similar age or with a younger woman, instead of remaining single. Further inspections of the data showed that by including the number of resident children only—instead of the overall respondent’s number of children, the positive effect vanishes. This is in line with previous work on the different role of resident and non-resident children for parents’ union formation (Beaujouan, 2012; Di Nallo, 2019; Sassler, 2010).

**Table 5:** Estimated coefficients and 95% confidence intervals for predictors of women’s entry into unions by partner’s age (homogamy = partner has similar age, hypergamy = man is older, hypogamy = woman is older)

Age assortative mating in wave 2 (Ref. single)	Coef.	Std. err.	95% Conf. Int	
*Age homogamy*				
Age	− 0.08	0.08	− 0.23	0.08
Age squared	0.00	0.00	0.00	0.00
Educational attainment (Ref. medium)				
Low	0.11	0.27	− 0.42	0.63
High	0.43	0.15	0.15	0.72
Number of children	− 0.14	0.12	− 0.37	0.09
Respondent union history (Ref. never in union)				
First union	0.52	0.21	0.11	0.93
Higher order union	1.15	0.31	0.54	1.76
Gender attitudes couple: age (Ref. non-egalitarian)				
Neutral	0.19	0.17	− 0.14	0.52
Egalitarian	0.36	0.18	0.02	0.71
NA	1.49	0.92	− 0.31	3.29
Gender attitudes couple: earnings (Ref. non-egalitarian)				
Neutral	− 0.29	0.20	− 0.67	0.10
Egalitarian	− 0.08	0.17	− 0.42	0.26
NA	− 1.87	1.07	− 3.97	0.23
Gender attitudes society (Ref. non-egalitarian)				
Neutral	0.05	0.25	− 0.43	0.54
Egalitarian	− 0.25	0.20	− 0.65	0.15
NA	0.75	0.81	− 0.85	2.34
Country (Ref. Bulgaria)				
Georgia	0.33	0.30	− 0.25	0.91
France	1.36	0.28	0.81	1.91
Hungary	1.06	0.28	0.50	1.61
Austria	1.77	0.28	1.22	2.31
Poland	1.09	0.26	0.58	1.60
Constant	− 0.90	1.11	− 3.07	1.27
*Age hypergamy*				
Age	0.02	0.05	− 0.08	0.12
Age squared	0.00	0.00	0.00	0.00
Educational attainment (Ref. medium)				
Low	− 0.02	0.16	− 0.34	0.30
High	0.09	0.10	− 0.12	0.29
Number of children	0.07	0.07	− 0.07	0.21
Respondent union history (Ref. never in union)				
First union	0.18	0.15	− 0.11	0.47
Higher order union	0.80	0.22	0.37	1.23
Gender attitudes couple: age (Ref. non-egalitarian)				
Neutral	− 0.16	0.11	− 0.38	0.06
Egalitarian	0.02	0.12	− 0.23	0.26
NA	− 0.13	0.78	− 1.66	1.40
Gender attitudes couple: earnings (Ref. non-egalitarian)				
Neutral	0.17	0.13	− 0.09	0.42
Egalitarian	− 0.18	0.12	− 0.42	0.07
NA	− 0.42	0.56	− 1.52	0.68
Gender attitudes society (Ref. non-egalitarian)				
Neutral	− 0.08	0.17	− 0.42	0.26
Egalitarian	− 0.02	0.14	− 0.29	0.25
NA	0.66	0.59	− 0.49	1.82
Country (Ref. Bulgaria)				
Georgia	− 0.75	0.16	− 1.07	− 0.43
France	0.39	0.16	0.09	0.70
Hungary	− 0.26	0.16	− 0.58	0.05
Austria	0.68	0.16	0.38	0.99
Poland	− 0.43	0.15	− 0.72	− 0.14
Constant	− 0.29	0.75	− 1.76	1.17
*Age hypogamy*				
Age	0.42	0.10	0.21	0.62
Age squared	− 0.01	0.00	− 0.01	0.00
Educational attainment (Ref. medium)				
Low	− 0.19	0.29	− 0.76	0.39
High	− 0.26	0.20	− 0.65	0.14
Number of children	0.06	0.10	− 0.13	0.25
Respondent union history (Ref. never in union)				
First union	0.33	0.24	− 0.15	0.81
Higher order union	0.96	0.31	0.35	1.58
Gender attitudes couple: age (Ref. non-egalitarian)				
Neutral	0.17	0.23	− 0.28	0.63
Egalitarian	0.55	0.23	0.10	1.00
NA	0.99	1.19	− 1.34	3.32
Gender attitudes couple: earnings (Ref. non-egalitarian)				
Neutral	− 0.38	0.26	− 0.88	0.12
Egalitarian	− 0.12	0.22	− 0.55	0.31
NA	− 1.08	1.13	− 3.29	1.13
Gender attitudes society (Ref. non-egalitarian)				
Neutral	− 0.11	0.33	− 0.76	0.54
Egalitarian	− 0.23	0.27	− 0.76	0.29
NA	NA	NA	NA	NA
Country (Ref. Bulgaria)				
Georgia	− 0.40	0.40	− 1.19	0.39
France	0.79	0.33	0.14	1.43
Hungary	0.64	0.32	0.01	1.27
Austria	1.20	0.33	0.56	1.84
Poland	0.43	0.32	− 0.20	1.05
Constant	− 9.23	1.61	− 12.38	− 6.07
Log likelihood	− 2935			
*N*	3503			

### Gender Egalitarian Attitudes and Educational Pairing

**Fig. 3:**
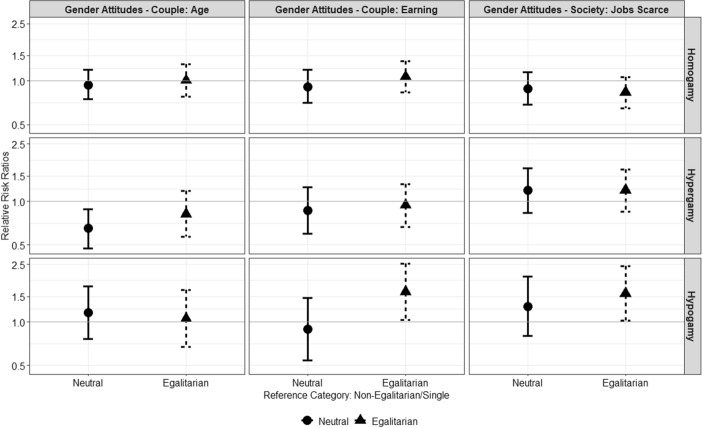
Relative risk ratios and 95% confidence intervals for gender-egalitarian attitudes as predictors of men’s entry into unions by partner’s education (homogamy = partner has similar educational level, hypergamy = man is more educated, hypogamy = woman is more educated)

Figure 3 shows the model estimates by gender-attitudes of the likelihood that a single man enters into a union with a woman equally educated as himself (homogamy), lower educated (hypergamy), higher educated (hypogamy) relative to remaining single. According to *H*2, egalitarian individuals would be more inclined to form hypogamous unions relative to non-egalitarian. In line with *H*2, results indicate that men who disagrees with the statements “If the woman earns more than the partner, it is not good for the relationship”, and “When jobs are scarce men have more right to the job than women” are about 60% more likely to form hypogamous unions instead of remaining single compared to men who agreed with those statements.

Furthermore, results show that individuals who hold neutral views about the age-difference between partners are less likely than individuals with non-egalitarian attitudes to form educational hypergamous unions than remaining single. The estimate relative to men holding egalitarian attitudes is also negative, but it is not statistically significant. The sign of these effects indicates that men with neutral and—to a lesser extent—egalitarian views related to partners’ age difference, compared to non-egalitarian men, are less likely to enter educational hypergamous unions, where the man is more educated than the woman, relatively to remain single. This finding recalls the finding from Fig. 1, where the outcome is age-hypergamy instead of educational hypergamy. Overall, findings seem to indicate that as men tend to hold egalitarian views, their mating behaviour drifts away from hypergamous mating pairings. Still, the evidence is weak because such effects do not show up for all the indicators of gender attitudes, but only for the one related to the age-difference between partners.

**Fig. 4:**
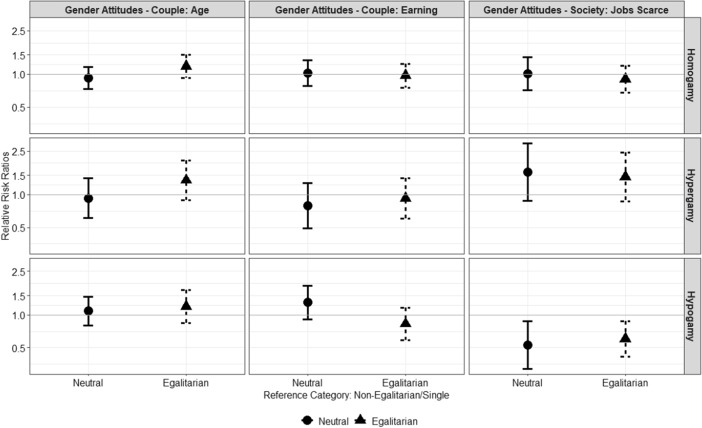
Relative risk ratios and 95% confidence intervals for gender-egalitarian attitudes as predictors of women’s entry into unions by partner’s education (homogamy = partner has similar educational level, hypergamy = man is more educated, hypogamy = woman is more educated)

Figure 4 shows results for the women sample. Contrary to the expectations, egalitarian women who disagreed with the statement “When jobs are scarce men have more right to the job than women” are less likely to form hypogamous unions instead of remaining single relatively to their non-egalitarian counterparts, i.e. women who agreed with that statement. It is worth reminding that women who agreed with this statement were very few, about 18% of the sample. These women represent a selective part of the sample, whereas the egalitarian group is larger and more heterogeneous.

As robustness check, I also run a multinomial model with partner’s educational level as outcome variable (results not shown, but available upon request) for both men and women separately. Results showed that egalitarian men are more likely to mate with a highly educated woman instead of remaining single relative to the non-egalitarian counterparts. Egalitarian women, instead, do not statistically differ from their non-egalitarian counterparts in the likelihood of partnering, independently from the partner’s educational level, relative to remaining single. The result of this robustness check is altogether in line with what has been already discussed.

## Discussion

It is argued that the diffusion of gender egalitarian attitudes will have positive effects on family outcomes, such as inhibiting union dissolution and enhancing parenthood (Goldscheider et al., 2015). Previous studies have analysed the role of gender egalitarian attitudes for family outcomes, e.g. entering a non-marital union, divorcing, having—or intend to have children (Greenstein, 1995; Kaufman, 2000; Puur et al., 2010; Aassve et al., 2015). It is still unclear, however, to what extent gender-egalitarian attitudes are associated with assortative mating, which in turn may also affect the stability and fertility of unions. This is an important gap, especially given the relevance of changes in assortative mating for family outcomes (Van Bavel, 2012).

This paper aimed to fill this gap by showing that gender egalitarian attitudes are also associated with the type of union that individuals tend to form, in terms of both age and educational differentials between partners. By pooling GGS data of six European countries and considering the two waves of GGS, I analysed the association between gender attitudes, measured at the time of first wave, and age and educational assortative mating, observed at the time of the second wave. Gender egalitarian attitudes were measured on single individuals using two statements regarding couple-relationships, and one statement related to the role of men and women in society. Hence, I tested hypotheses *H*1 and *H*2.

According to *H*1, egalitarian men and women are more likely to form *age hypogamous unions*, where the woman is older than the man, relative to their non-egalitarian counterparts. Similarly, following *H*2*,* I expected that both egalitarian men and women are more likely to form *educationally hypogamous unions*, where the woman is more educated than the man, relative to non-egalitarian individuals.

I found supporting evidence for *H*1: models’ results showed that both single egalitarian men and women, who disagreed with the statement “In a couple it is better for the man to be older than the woman”, are more likely to form unions where the woman is older than the man compared to non-egalitarian individuals. Additionally with regard to educational assortative mating, I found only partial evidence supporting *H*2. In line with *H*2, I found that single egalitarian men are more likely than single non-egalitarian men to form unions where the woman is more educated than themselves. Contrary to *H*2, I found that single egalitarian women, who disagreed with the statement “When jobs are scarce men have more right to the job than women”, are less likely than single non-egalitarian women to form educationally hypogamous unions.

Additionally, I found that men who tended towards egalitarian views regarding the ideal age-difference between partners were less likely to form hypergamous unions (both age- and educational hypergamy) compared to their non-egalitarian peers. Despite the evidence is relatively weak, nothing in this direction was observed for women.

Overall, these results highlight the importance of gender egalitarian attitudes for patterns of union formation. Gender-egalitarian attitudes, however, were not always predictive of mating behaviour in the direction expected. In particular, when the independent variable explicitly matched the outcome, such as in the case of gender-egalitarian attitudes related to partners’ age-difference which were predictive of age-mating patterns, attitudes predicted behaviour for both men and women. When the independent variable was only indirectly linked to the outcome, the relation between gender attitudes and mating behaviours was not consistent between genders. Evidence presented here shows that for men with a low or medium level of education holding egalitarian views may increase their chances to avoid singlehood. This, however, does not hold for women with at least a medium educational level. Regardless of gender attitudes, women with a medium or high level of education may still prefer to mate with someone who has good economic prospects (Eastwick et al., 2006; Van Bavel, 2021; Zentner & Eagly, 2015). Thus, attitudes about the ideal difference in earnings between the partners and about the role of men and women in society might not necessarily reflect women’s educational mating outcomes.

Some important limitations of this study should be mentioned. First, due to the low number of events and small sample size, it was not possible to analyse countries separately. This is unfortunate because the countries considered are at different stages of the Gender Revolution, which implies different levels of gender-equality in the public and private spheres. As Bellani et al. (2017) showed, cross-country variation in the probability to remain single is associated with the diffusion of gender egalitarian attitudes. To check whether results were sensitive to the sample composition, I run models dropping one country at the time and results did not lead to different conclusions (see Electronic Supplementary Resources). In the future, it would be interesting to test the role of context in the association between gender role attitudes and patterns of assortative mating.

Next, following Davis and Greenstein (2009), the conceptualization of gender-role attitudes included statements relative to the primacy of breadwinner role, and the belief in the gendered separate spheres. Due to lack of information, I could not include statements regarding attitudes towards the involvement of man in household chores. The attitudes towards a participation of men in household tasks are regarded as indicator of progress to the second phase of the Gender Revolution. An aspect that future studies could consider if comparable data will be available.

Finally, due to the small number of events it was not possible to do an analysis combining age and educational assortative mating patterns. As a result, *age-hypogamous* unions may well be hypergamous or homogamous in terms of education, income, occupational prestige etc. Similarly, *educationally hypogamous* unions could be hypergamous or homogamous in other aspects. The statement regarding partners’ age was especially predictive for age-assortative mating patterns, whereas statements about partners’ earnings and the right to a job for men and women were exclusively predictive of educational assortative mating. As a result, among the newly formed *hypogamous* couples compensating behaviours may take place, shifting the power towards the partner with more socio-economic resources (Bozon, 1991). Whether compensating behaviours take place, and to what extent the diffusion of gender-egalitarian attitudes somewhat inhibit such behaviours at the individual and societal level is a question that should be answered in the future.

Despite the limitations, this paper contributes to previous work about mating selection processes, and more generally about the role of gender attitudes for family outcomes. The evidence presented in this paper points towards the fact that gender-attitudes may be predictive of mating behaviour, especially when the measured attitudes match closely the type of mating outcome.

Moreover, this study highlights similarities and differences in men and women’s mating outcomes related to their gender attitudes. A convergence in mating preferences between men and women would imply that men are more and more willing to choose more educated—possibly working—partners. According to the results shown, this is the case for egalitarian men, who are more likely to form *hypogamous* unions than non-egalitarian men. In this sense, men’s mating behaviour may become similar to women’s behaviour. Instead, there is no evidence that women, driven by egalitarian views, tend to mate with less educated men. Further studies that can corroborate these findings are needed.

Selection processes occurring at the time of union formation have notable consequences for family outcomes, especially regarding fertility decision-making processes (Basu, 2002). Couples’ disagreement on family goals is among the main reason fertility is below desired levels (Voas, 2003). The study of mating selection processes and its determinants would give us tools to further understand mechanisms of fertility decision-making, and possibly to better theorize about the future of family outcomes.

## Appendix

See Tables 4, 5,

**Table 6:** Estimated coefficients and 95% confidence intervals for predictors of men’s entry into unions by partner’s education (homogamy = both same education level, hypergamy = man more educated, hypogamy = woman more educated)

Educational assortative mating in wave 2 (Ref. single)	Coef	Std. Err.	95% Conf. Int	
*Homogamy*				
Age	0.27	0.06	0.15	0.39
Age squared	− 0.01	0.00	− 0.01	0.00
Educational attainment (Ref. medium)				
Low	− 0.05	0.15	− 0.34	0.24
High	0.36	0.11	0.15	0.57
Gender attitudes couple: age (Ref. Non-egalitarian)				
Neutral	− 0.06	0.12	− 0.29	0.18
Egalitarian	0.01	0.13	− 0.25	0.27
NA	0.41	0.56	− 0.69	1.51
Gender attitudes couple: earnings (Ref. Non-egalitarian)				
Neutral	− 0.09	0.13	− 0.35	0.18
Egalitarian	0.07	0.13	− 0.18	0.32
NA	− 0.68	0.48	− 1.61	0.25
Gender attitudes society (Ref. Non-egalitarian)				
Neutral	− 0.12	0.13	− 0.38	0.14
Egalitarian	− 0.18	0.12	− 0.43	0.06
NA	0.26	0.52	− 0.76	1.28
Number of children	0.21	0.10	0.01	0.42
Respondent union history (Ref. Never in union)				
First union	0.44	0.17	0.12	0.77
Higher order union	0.29	0.29	− 0.28	0.86
Country (Ref. Bulgaria)				
Georgia	0.59	0.17	0.25	0.93
France	1.14	0.20	0.74	1.54
Hungary	1.30	0.16	0.99	1.61
Austria	1.67	0.18	1.31	2.03
Poland	− 0.02	0.19	− 0.39	0.36
Constant	− 5.34	0.87	− 7.03	− 3.64
*Hypergamy*				
Age	− 0.04	0.08	− 0.20	0.11
Age Squared	0.00	0.00	0.00	0.00
Educational attainment (Ref. medium)				
Low	NA	NA	NA	NA
High	0.98	0.15	0.69	1.27
Gender attitudes couple: age (Ref. non-egalitarian)				
Neutral	− 0.43	0.16	− 0.75	− 0.12
Egalitarian	− 0.20	0.19	− 0.56	0.17
NA	− 1.25	1.18	− 3.57	1.06
Gender attitudes couple: earnings (Ref. non-egalitarian)				
Neutral	− 0.14	0.19	− 0.51	0.23
Egalitarian	− 0.06	0.18	− 0.41	0.28
NA	0.27	0.71	− 1.12	1.65
Gender attitudes society (Ref. non-egalitarian)				
Neutral	0.18	0.18	− 0.18	0.53
Egalitarian	0.18	0.17	− 0.16	0.51
NA	0.56	0.85	− 1.10	2.22
Number of children	0.41	0.14	0.14	0.67
Respondent union history (Ref. never in union)				
First union	0.62	0.24	0.15	1.08
Higher order union	0.97	0.41	0.17	1.77
Country (Ref. Bulgaria)				
Georgia	− 1.54	0.22	− 1.97	− 1.12
France	− 1.21	0.28	− 1.76	− 0.66
Hungary	− 1.18	0.20	− 1.58	− 0.78
Austria	− 0.36	0.22	− 0.80	0.08
Poland	− 2.54	0.31	− 3.16	− 1.93
Constant	− 0.07	1.12	− 2.27	2.13
*Hypogamy*				
Age	0.44	0.11	0.23	0.65
Age Squared	− 0.01	0.00	− 0.01	0.00
Educational attainment (Ref. medium)				
Low	0.88	0.19	0.52	1.25
High	NA	NA	NA	NA
Gender attitudes couple: age (Ref. non-egalitarian)				
Neutral	0.15	0.21	− 0.27	0.57
Egalitarian	0.05	0.23	− 0.40	0.51
NA	0.31	1.16	− 1.96	2.57
Gender attitudes couple: earnings (Ref. non-egalitarian)				
Neutral	− 0.12	0.25	− 0.61	0.38
Egalitarian	0.48	0.23	0.03	0.93
NA	− 1.39	1.09	− 3.53	0.74
Gender attitudes society (Ref. non-egalitarian)				
Neutral	0.25	0.24	− 0.22	0.72
Egalitarian	0.45	0.22	0.02	0.89
NA	0.12	1.12	− 2.06	2.31
Number of children	0.19	0.15	− 0.11	0.50
Respondent union history (Ref. never in union)				
First union	0.24	0.28	− 0.32	0.79
Higher order union	0.76	0.43	− 0.09	1.60
Country (Ref. Bulgaria)				
Georgia	1.32	0.30	0.72	1.91
France	1.39	0.33	0.73	2.04
Hungary	1.30	0.27	0.76	1.84
Austria	1.12	0.34	0.45	1.78
Poland	0.24	0.34	− 0.42	0.90
Constant	− 9.62	1.56	− 12.68	− 6.56
Log likelihood	− 2896			
*N*	3623			

^6^,

**Table 7:** Estimated coefficients and 95% confidence intervals for predictors of women’s entry into unions by partner’s education (homogamy = both same education level, hypergamy = man more educated, hypogamy = woman more educated)

Educational assortative mating in wave 2 (Ref. single)	Coef.	Std. err.	95% Conf. int	
*Homogamy*				
Age	0.12	0.06	0.01	0.22
Age squared	0.00	0.00	− 0.01	0.00
Educational attainment (Ref. medium)				
Low	− 0.06	0.18	− 0.41	0.29
High	0.05	0.10	− 0.16	0.25
Gender attitudes couple: age (Ref. non-egalitarian)				
Neutral	− 0.08	0.12	− 0.31	0.15
Egalitarian	0.17	0.13	− 0.08	0.41
NA	0.27	0.70	− 1.09	1.64
Gender attitudes couple: earnings (Ref. non-egalitarian)				
Neutral	0.02	0.13	− 0.24	0.29
Egalitarian	− 0.03	0.13	− 0.28	0.22
NA	− 0.53	0.49	− 1.50	0.43
Gender attitudes society (Ref. non-egalitarian)				
Neutral	0.01	0.18	− 0.34	0.36
Egalitarian	− 0.10	0.15	− 0.39	0.18
NA	0.49	0.58	− 0.64	1.63
Number of children	− 0.03	0.07	− 0.18	0.12
Respondent union history (Ref. never in union)				
First union	0.26	0.15	− 0.02	0.55
Higher order union	0.84	0.22	0.42	1.27
Country (Ref. Bulgaria)				
Georgia	0.14	0.19	− 0.24	0.52
France	1.14	0.19	0.78	1.50
Hungary	1.26	0.17	0.92	1.60
Austria	1.62	0.18	1.26	1.98
Poland	0.22	0.18	− 0.14	0.58
Constant	− 2.57	0.80	− 4.14	− 1.01
*Hypergamy*				
Age	0.03	0.09	− 0.15	0.20
Age Squared	0.00	0.00	0.00	0.00
Educational attainment (Ref. medium)				
Low	0.88	0.20	0.48	1.27
High	NA	NA	NA	NA
Gender attitudes couple: age (Ref. non-egalitarian)				
Neutral	− 0.08	0.21	− 0.49	0.34
Egalitarian	0.30	0.22	− 0.12	0.72
NA	0.12	1.05	− 1.94	2.18
Gender attitudes couple: earnings (Ref. non-egalitarian)				
Neutral	− 0.24	0.24	− 0.71	0.24
Egalitarian	− 0.08	0.21	− 0.50	0.34
NA	− 0.84	0.84	− 2.49	0.81
Gender attitudes society (Ref. non-egalitarian)				
Neutral	0.48	0.31	− 0.13	1.08
Egalitarian	0.37	0.26	− 0.15	0.88
NA	1.53	0.81	− 0.06	3.12
Number of children	− 0.02	0.10	− 0.22	0.17
Respondent union history (Ref. never in union)				
First union	0.37	0.25	− 0.11	0.85
Higher order union	0.62	0.34	− 0.04	1.28
Country (Ref. Bulgaria)				
Georgia	0.71	0.32	0.08	1.34
France	1.48	0.30	0.90	2.06
Hungary	0.96	0.31	0.36	1.56
Austria	1.78	0.29	1.22	2.35
Poland	− 0.17	0.36	− 0.89	0.54
Constant	− 2.98	1.32	− 5.56	− 0.39
*Hypogamy*				
Age	− 0.03	0.08	− 0.17	0.12
Age squared	0.00	0.00	0.00	0.00
Educational attainment (Ref. medium)				
Low	NA	NA	NA	NA
High	1.24	0.15	0.95	1.52
Gender attitudes couple: age (Ref. non-egalitarian)				
Neutral	0.08	0.15	− 0.22	0.38
Egalitarian	0.18	0.18	− 0.17	0.52
NA	1.28	0.91	− 0.50	3.07
Gender attitudes couple: earnings (Ref. non-egalitarian)				
Neutral	0.26	0.18	− 0.10	0.61
Egalitarian	− 0.19	0.17	− 0.53	0.15
NA	0.36	0.66	− 0.93	1.65
Gender attitudes society (Ref. non-egalitarian)				
Neutral	− 0.64	0.25	− 1.14	− 0.14
Egalitarian	− 0.51	0.19	− 0.88	− 0.13
NA	− 2.00	1.28	− 4.50	0.51
Number of children	0.30	0.11	0.08	0.52
Respondent union history (Ref. never in union)				
First union	0.28	0.21	− 0.13	0.70
Higher order union	1.23	0.32	0.61	1.85
Country (Ref. Bulgaria)				
Georgia	− 2.20	0.26	− 2.70	− 1.70
France	− 0.93	0.23	− 1.38	− 0.48
Hungary	− 0.54	0.19	− 0.91	− 0.17
Austria	− 0.57	0.23	− 1.02	− 0.11
Poland	− 1.68	0.22	− 2.11	− 1.25
Constant	0.12	1.07	− 1.97	2.21
Log likelihood	− 2841			
*N*	3522			

^7^.

## References

[CR1] Aassve A, Fuochi G, Mencarini L (2014). Desperate housework: Relative resources, time availability, economic dependency, and gender ideology across Europe. Journal of Family Issues.

[CR2] Aassve A, Fuochi G, Mencarini L, Mendola D (2015). What is your couple type? Gender ideology, housework sharing, and babies. Demographic Research.

[CR3] Agresti A (2002). Logit models for multinomial responses. Categorical data analysis.

[CR4] Basu AM (2002). Why does education lead to lower fertility? A critical review of some of the possibilities. World Development.

[CR6] Beaujouan É (2012). Repartnering in France: The role of gender, age and past fertility. Advances in Life Course Research.

[CR7] Becker GS (1991). A treatise on the family.

[CR8] Bellani D, Esping-Andersen G, Nedoluzhko L (2017). Never partnered: A multilevel analysis of lifelong singlehood. Demographic Research.

[CR9] Blossfeld, H. P., & Timm, A. (2003). *Who marries whom? Educational systems as marriage markets in modern societies*. Springer.

[CR10] Bozon M (1991). Women and the age gap between spouses: An accepted domination?. Population. English Selection.

[CR11] Brines J (1994). Economic dependency, gender, and the division of labour at home. American Journal of Sociology.

[CR12] Burgess EW, Wallin P (1943). Homogamy in social characteristics. American Journal of Sociology.

[CR13] Buss D (2000). Number of children desired and preferred spousal age difference: Context-specific mate preference patterns across 37 cultures. Evolution and Human Behaviour.

[CR14] Buss DM, Abbott M, Angleitner A, Asherian A, Biaggio A, Blanco-Villasenor A (1990). International preferences in selecting mates: A Study of 37 Cultures. Journal of Cross-Cultural Psychology.

[CR15] Buss DM, Shackelford TK, Kirkpatrick LA, Larsen RJ (2001). A half century of mate preferences: The cultural evolution of values. Journal of Marriage and Family.

[CR16] Davis SN, Greenstein TN (2009). Gender ideology: Components, predictors, and consequences. Annual Review of Sociology.

[CR17] De Hauw Y, Grow A, Van Bavel J (2017). The reversed gender gap in education and assortative mating in Europe. European Journal of Population.

[CR18] Di Nallo A (2019). Gender gap in repartnering: The role of parental status and custodial arrangements. Journal of Marriage and Family.

[CR19] Eagly AH, Wood W (1999). The origins of sex differences in human behaviour: Evolved dispositions versus social roles. American Psychologist.

[CR20] Eastwick PW, Eagly AH, Glick P, Johannesen-Schmidt MC, Fiske ST, Blum AMB, Eckes T (2006). Is traditional gender ideology associated with sex-typed mate preferences? A test in nine nations. Sex Roles.

[CR21] Esping-Andersen, G. (2009). *Incomplete revolution: Adapting welfare states to women's new roles*. Polity.

[CR22] Esping-Andersen G, Billari FC (2015). Re-theorizing family demographics. Population and Development Review.

[CR23] Esteve A, García-Román J, Permanyer I (2012). The gender-gap reversal in education and its effect on union formation: The end of hypergamy?. Population and Development Review.

[CR24] Esteve A, Schwartz CR, Van Bavel J, Permanyer I, Klesment M, Garcia J (2016). The end of hypergamy. Population and Development Review.

[CR25] Frye M, Urbina DR (2019). Fearing such a lady: University expansion, underemployment, and the hypergamy ideal in Kampala, Uganda. Journal of Family Issues..

[CR26] Gauthier A, Cabaço S, Emery T (2018). Generations and gender survey study profile. Longitudinal and Life Course Studies.

[CR27] Goldscheider F, Bernhardt E, Lappegard T (2015). The gender revolution: Understanding changing family and demographic behaviour. Population and Development Review.

[CR28] Goldscheider F, Kaufman G, Sassler S (2009). Navigating the “new” marriage market. Journal of Family Issues.

[CR29] Goldscheider F, Oláh LS, Puur A (2010). Reconciling studies of mens gender attitudes and fertility. Demographic Research.

[CR30] Greenstein TN (1995). Gender ideology, marital disruption, and the employment of married-women. Journal of Marriage and the Family.

[CR31] Grow A, Van Bavel J (2015). Assortative mating and the reversal of gender inequality in education in Europe: An agent-based model. PLoS ONE.

[CR32] Hart RK (2019). Union histories of dissolution: What can they say about childlessness?. European Journal of Population.

[CR33] Kaufman G (2000). Do gender role attitudes matter? Family formation and dissolution among traditional and egalitarian men and women. Journal of Family Issues.

[CR34] Klesment M, Van Bavel J (2017). The reversal of the gender gap in education, motherhood, and women as main earners in Europe. European Sociological Review.

[CR35] Kolk M (2015). Age differences in Unions: Continuity and divergence among swedish couples between 1932 and 2007. European Journal of Population.

[CR36] Meeussen L, Van Laar C, Verbruggen M (2019). Looking for a family man? Norms for men are toppling in heterosexual relationships. Sex Roles.

[CR37] Nitsche N, Matysiak A, Bavel JV, Vignoli D (2018). Partners’ educational pairings and fertility across Europe. Demography.

[CR38] Oppenheimer V (1988). A theory of marriage timing. American Journal of Sociology.

[CR39] Press JE (2004). Cute butts and housework: A gynocentric theory of assortative mating. Journal of Marriage and Family.

[CR40] Rindfuss RR, Brewster KL, Kavee AL (1996). Women, work, and children: Behavioral and attitudinal change in the United States. Population and Development Review.

[CR41] Sassler S (2010). Partnering across the life course: Sex, relationships, and mate selection. Journal of Marriage and Family.

[CR42] Schwartz CR, Han H (2014). The reversal of the gender gap in education and trends in marital dissolution. American Sociological Review.

[CR43] Theunis L, Schnor C, Willaert D, Van Bavel J (2018). His and her education and marital dissolution: Adding a contextual dimension. European Journal of Population.

[CR44] Thornton A, Young-DeMarco L (2001). Four decades of trends in attitudes toward family issues in the United States: The 1960s through the 1990s. Journal of Marriage and Family.

[CR45] Trimarchi A, Van Bavel J (2020). Partners’ educational characteristics and fertility: Disentangling the effects of earning potential and unemployment risk on second births. European Journal of Population.

[CR5] Van Bavel, J. (2012). The reversal of gender inequality in education, union formation and fertility in Europe. *Vienna Yearbook of Population Research*, no. June 2012: 1–40.

[CR46] Van Bavel, J. (2021). Partner choice and partner markets. In Norbert F. Schneider & Michaela Kreyenfeld (Eds.), *Research handbook on the sociology of the family* (pp. 219–231). Elgar.

[CR47] Van Bavel J, Schwartz CR, Esteve A (2018). The reversal of the gender gap in education and its consequences for family life. Annual Review of Sociology.

[CR48] Van De Kaa DJ (1987). Europe’s second demographic transition. Population Bulletin.

[CR49] Van Houdt K, Poortman A-R (2018). Joint lifestyles and the risk of union dissolution: Differences between marriage and cohabitation. Demographic Research.

[CR50] Vikat A, Spéder Z, Beets G, Billari F, Bühler C, Desesquelles A, Fokkema T (2007). Generations and gender survey (GGS). Demographic Research.

[CR51] Voas D (2003). Conflicting preferences: A reason fertility tends to be too high or too low. Population and Development Review.

[CR52] Zentner M, Eagly AH (2015). A sociocultural framework for understanding partner preferences of women and men: Integration of concepts and evidence. European Review of Social Psychology.

